# FAPI PET for monitoring of rheumatological treatment in multifocal peritoneal nodular fibrosis: a case study

**DOI:** 10.1007/s00259-024-07052-7

**Published:** 2025-01-06

**Authors:** Lena M. Unterrainer, Stephan T. Ledderose, Sophie C. Kunte, Johannes Toms, Clemens C. Cyran, Adrien Holzgreve, Marcus Unterrainer, Hendrik Schulze-Koops

**Affiliations:** 1 Department of Nuclear Medicine, University Hospital, LMU Munich, Marchioninistrasse 15, 81377 Munich, Germany; 2 Ahmanson Translational Theranostics Division, Department of Molecular and Medical Pharmacology, David Geffen School of Medicine, UCLA, Los Angeles, CA USA; 3 Bavarian Cancer Research Center (BZKF), Partner Site Munich, Munich, Germany; 4 Institute of Pathology, LMU Munich, Munich, Germany; 5 Department of Radiology, University Hospital, LMU Munich, Munich, Germany; 6 Die Radiologie, Munich, Germany; 7 Division of Rheumatology and Clinical Immunology, Department of Internal Medicine IV, LMU Munich, Munich, Germany

Here we report a 71-year-old male patient with ANCA-negative vasculitis and rapidly progressive glomerulonephritis presenting with increasing fatigue, weight loss of 10 kg over 3 months, and elevated inflammatory biomarkers (CRP: 7.2 mg/dl). A contrast-enhanced CT showed diffuse soft-tissue proliferation in the perirenal region and the omentum majus with multiple nodular implants in the peritoneal fatty tissue. A biopsy from the infiltrated omentum majus revealed predominantly histiocytic serositis with multinodular fibrosis on H&E. Immunohistochemical analyses showed scattered CD38-positive plasma cells and a small number of IgG4-positive plasma cells (Figure).

An ^18^F-FAPI-74 PET/CT was performed prior to systemic anti-inflammatory therapy comprising increased uptake in the multifocal peritoneal nodular implants (Figure: *) and in the aortic arch (Figure, arrow). Five weeks after the initiation of oral treatment with prednisolone (initially 1 mg/kg bodyweight with a taper to ½ mg/kg bodyweight at week five), the patient underwent a follow-up ^18^F-FAPI-74 PET/CT (follow-up 1) with significantly reduced FAPI-uptake in the aortic arch as well as in the peritoneal nodules, despite only slightly decreased morphological appearance of the peritoneal implants, but significantly improved general condition.


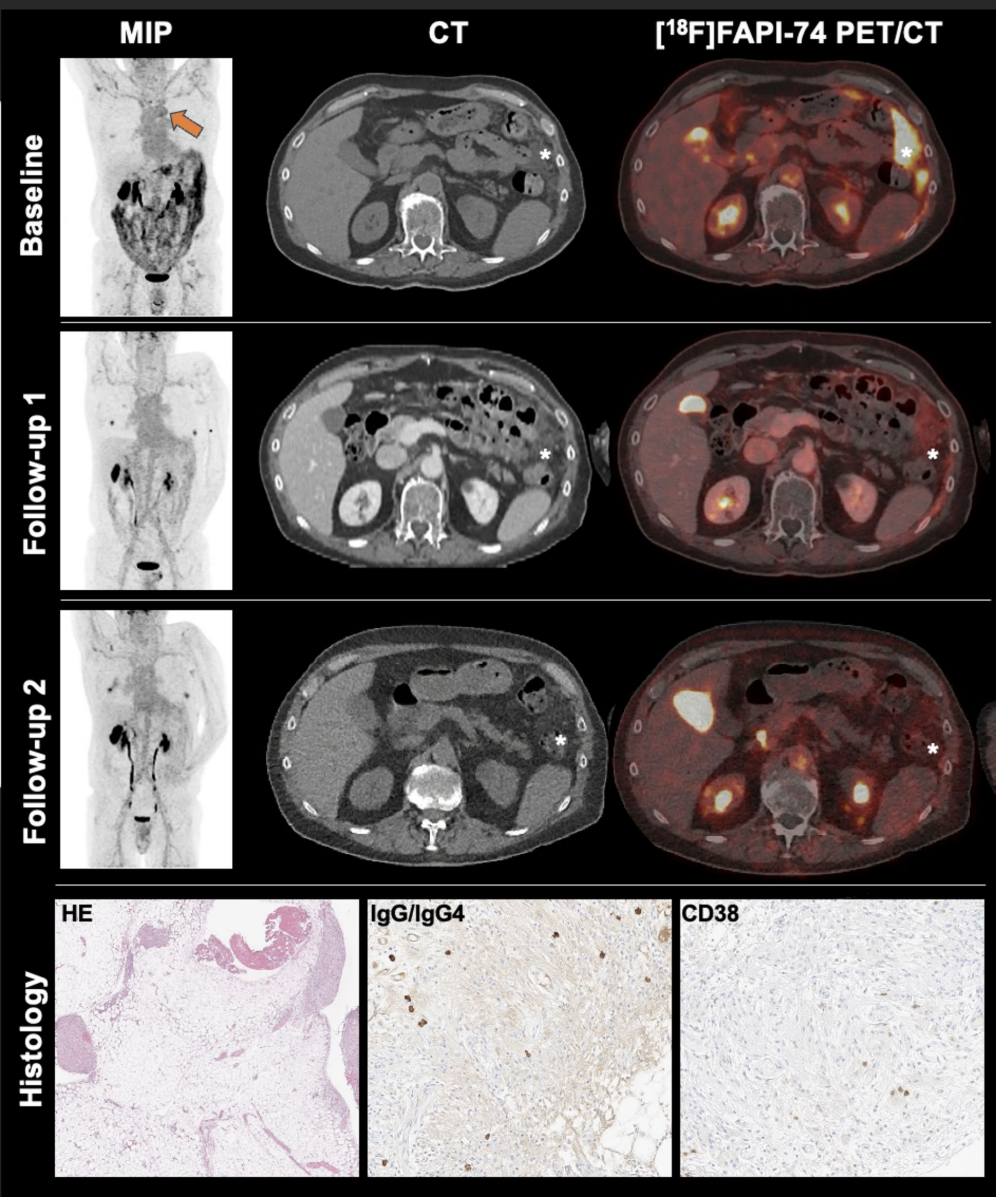


A further ^18^F-FAPI-74 PET/CT three months after the initiation of glucocorticoids showed a no further change regarding FAPI-uptake (follow-up 2). This case illustrates the potential of imaging FAP-associated changes in rheumatoid patients undergoing systemic anti-inflammatory therapy, where changes in PET imaging might depict disease activity earlier than morphological imaging and might predict response to therapy [1].

## Data Availability

The dataset generated for this case report are available from the corresponding author on reasonable request.
